# Dual role of CsrA in regulating the hemolytic activity of *Escherichia coli* O157:H7

**DOI:** 10.1080/21505594.2022.2073023

**Published:** 2022-05-24

**Authors:** Zhibin Sun, Ning Zhou, Wenting Zhang, Yan Xu, Yu-Feng Yao

**Affiliations:** a Laboratory of Bacterial Pathogenesis, Department of Microbiology and Immunology, Institutes of Medical Sciences, Shanghai Jiao Tong University School of Medicine, Shanghai, China; b Jiangsu Province Key Laboratory of Oral Diseases, Nanjing Medical University, Nanjing, Jiangsu, China; c Department of Infectious Diseases, Shanghai Ruijin Hospital, Shanghai, China; d Shanghai Key Laboratory of Emergency Prevention, Diagnosis and Treatment of Respiratory Infectious Diseases, Shanghai, China

**Keywords:** *Escherichia coli* O157:H7, hemolysis, CsrA/RsmA, virulence, protein–RNA interaction

## Abstract

Post-transcriptional global carbon storage regulator A (CsrA) is a sequence-specific RNA-binding protein involved in the regulation of multiple bacterial processes. Hemolysin is an important virulence factor in the enterohemorrhagic *Escherichia coli* O157:H7 (EHEC). Here, we show that CsrA plays a dual role in the regulation of hemolysis in EHEC. CsrA significantly represses plasmid-borne enterohemolysin (EhxA)-mediated hemolysis and activates chromosome-borne hemolysin E (HlyE)-mediated hemolysis through different mechanisms. RNA structure prediction revealed a well-matched stem-loop structure with two potential CsrA binding sites located on the 5' untranslated region (UTR) of *ehxB*, which encodes a translocator required for EhxA secretion. CsrA inhibits EhxA secretion by directly binding to the RNA leader sequence of *ehxB* to repress its expression in two different ways: CsrA either binds to the Shine–Dalgarno sequence of *ehxB* to block ribosome access or to *ehxB* transcript to promote its mRNA decay. The predicted CsrA-binding site 1 of *ehxB* is essential for its regulation. There is a single potential CsrA-binding site at the 5'-end of the *hlyE* transcript, and its mutation completely abolishes CsrA-dependent activation. CsrA can also stabilize *hlyE* mRNA by directly binding to its 5' UTR. Overall, our results indicate that CsrA acts as a hemolysis modulator to regulate pathogenicity under certain conditions.

## Introduction

Hemolysins are among the major virulence factors identified in various bacterial pathogens [1]. *Escherichia coli* hemolysins can be divided into three types: *E. coli* α-hemolysin (HlyA), enterohemolysin (EhxA), and hemolysin E (HlyE) [2]. Hemolysins EhxA and HlyE are present in the enterohemorrhagic *E. coli* (EHEC). EhxA shares high similarity with HlyA, which is a key virulence factor encoded on the chromosome and plasmid of pathogenic *E. coli* strains [3,4]. It belongs to the RTX family of proteins and is encoded on the virulence plasmids of typical EHEC strains, such as O157:H7 [4]. EhxA is a potential virulence factor that significantly correlates with severe diseases, such as hemolytic uremic syndrome (HUS) and hemorrhagic colitis (HC) [5]. However, EhxA normally exhibits weaker hemolytic activity than HlyA [6].

The synthesis of EhxA is directed by the *ehxCABD* operon, which shares nearly 60% identity with the *hlyCABD* operon [7]. Like *hlyCABD*, *ehxA* and *ehxC* encode a hemolytic toxin and an activator involved in the posttranslational modification process of EhxA, respectively [4,5]. The secretion structure is composed of three proteins: an ABC transporter, EhxB; a membrane fusion protein (MFP), EhxD; and an outer membrane protein (OMP), TolC. Unlike EhxB and EhxD, TolC is not a plasmid-coded protein, and it involved in many cellular processes [8]. The inner membrane protein complex EhxB-EhxD and TolC form a nanomachine that exports EhxA through the bacterial cell wall [5,8]. Although both operons form similar type 1 secretion systems (T1SSs) that deliver effectors to the extracellular space, the biochemical properties of the hemolysins produced by each operon are different [9]. EhxA can be found either free or in association with outer membrane vesicles released by EHEC strains. Most free-EhxA targets cell membranes and damages microvascular endothelial cells via pore formation [10]. The outer membrane vesicle-associated EhxA targets mitochondria, causing apoptosis via the capase-9-dependent pathway [5]. EhxA is also used as a phenotypic marker to identify typical EHEC strains [11].

The *ehxCABD* operon of EHEC O157:H7 is located on the non-transmissible plasmid pO157 and is influenced by several regulators at the transcriptional level. Regulators such as the GrlA-GrlR regulatory system and Ler, which are encoded by the locus of enterocyte effacement (LEE) island can affect hemolysin expression [12]. Deletion of *grlR* or overexpression of GrlA significantly increases the lytic activity of Ehx, thus indicating that GrlA is a positive regulator of Ehx expression, while GrlR is a negative regulator of Ehx expression at the transcriptional level [13]. Ler is an expression activator of most LEE genes and can activate Ehx expression, either as a GrlA activator or in a GrlA-independent manner [13]. This GrlA-independent EhxA expression may be facilitated via the regulation of *ehxC* by Ler, which may directly interact with the regulatory region of *ehxC* [14]. LrhA, a non-LEE-encoded regulator, upregulates the expression of LEE genes and can also activate EhxA expression [14]. Li et al. suggested that the expression of proteins by the *ehxCABD* operon could be regulated by the global transcriptional regulation factors H-NS, DsrA, and σ factor RpoS [15]. The expression of proteins by the *ehxCABD* operon was silenced by H-NS, which could be antagonized by DsrA [15]. A mutation in *rpoS* completely abolished *ehxA* transcription, indicating that RpoS is an essential regulator of Ehx expression [16]. The regulation of Ehx expression therefore seems to be a complex, multifactorial process. However, examining the interactions between regulators and their influence on the *ehxCABD* operon can help to uncover the clinical importance of EhxA.

HlyE is a pore-forming toxin that has been identified in Enterobacteriaceae such as *E. coli*, *Shigella flexneri*, and *Salmonella* Typhi [17]. It is unrelated to the *E. coli* hemolysins HlyA and EhxA [18,19]. Unlike HlyA and EhxA, which are synthesized as soluble protoxins that require proteolytic and posttranslational modification processes to become active toxins, HlyE does not require posttranslational processing [17]. Previous studies have revealed that the genome of *Salmonella* Typhi, causing typhoid fever and that of *S. flexneri*, causing dysentery, encode proteins that are highly homologous to HlyE [17]. HlyE plays an essential role in epithelial cell invasion and deep organ infection and can thus be regarded as a significant component of these pathogens’ armory of toxins [20].

The global carbon storage regulator (Csr) system plays an important role in controlling many cellular processes, such as carbon metabolism, motility, stress response, quorum sensing, biofilm formation, iron storage, and pathogenicity [21–29]. Csr is widely distributed among gram-negative bacteria, and it also occurs in the gram-positive bacterium *Bacillus subtilis* [30]. Homologous regulatory factors of Csr are known to be repressors of secondary metabolites (Rsm) [30]. The four major components of Csr in *E. coli* are as follows: CsrA is a homo-dimeric RNA-binding protein; CsrB and CsrC are two small RNAs acting as CsrA antagonists; and CsrD is a factor targeting CsrB and CsrC specifically for degradation by RNase E [31–33]. CsrA, a key component of the Csr system, preferentially binds to mRNA in the 5' untranslated region (5' UTR) or the early coding region [30]. The specific binding motif GGA is normally located in the single stranded loop of a hairpin structure [34]. The CsrA binding to these sites can therefore affect translation, RNA stability, and RNA structure for Rho-dependent transcription termination [35–39]. Several studies have revealed that CsrA is not only capable of directly targeting multiple RNA but also of controlling other regulators, confirming its crucial role in bacterial physiology and virulence [24,40].

The present study investigated the dual role of CsrA in hemolysis regulation. CsrA can be considered as contributing to the EHEC pathogenicity by facilitating the coordination of virulence factors.

## Materials and methods

### Bacterial strains and culture conditions

Bacterial strains (Supplementary material, Table S1) were grown routinely in a Luria-Bertani (LB) medium at pH 7.4 and temperature 37°C with shaking (250 rpm). Antibiotics were added at the following final concentrations: tetracycline, 10 μg/mL; ampicillin, 100 μg/mL; chloramphenicol, 15 μg/mL; and kanamycin, 50 μg/mL. All chemical reagents were purchased from Sigma-Aldrich (St. Louis, MI, USA), unless stated otherwise.

### Construction of plasmids and mutant strains

All primers used in this study (Supplementary material, Table S2) were synthesized by Sangon Biotech Co., Ltd (Shanghai, China). All mutants were constructed using the λ Red recombinase system [41]. A truncated *csrA* strain exhibiting reduced RNA-binding affinity and partial activity was constructed using the method described by Wang et al. [42]. The truncation of *csrA* was confirmed by PCR. For *csrA* complementation, *csrA* and its upstream 500-bp sequence were amplified using primers 184-CsrA-F and 184-CsrA-R. The amplified DNA fragment was then inserted into the plasmid cloning vector pACYC184 at restriction sites *Nco*I and *Sca*I to obtain the plasmid pCSRA. For EhxA overexpression, *ehxA* was PCR amplified using primers 80-ehxA-F and 80-ehxA-R. The obtained DNA fragment was inserted into the plasmid cloning vector pQE80-YX1 at restriction sites *Afe*I and *Fse*I to construct plasmid pQE-*ehxA*.

EHEC *ΔlacI-Z*, a β-galactosidase-defective strain, was constructed following the method described by Jackson et al. [26]. To construct *lacZ* reporter fusions, the plasmid cloning vector pACYC184 was employed. The *lacZ* gene, containing *Eco*RI and *Sca*I restriction sites, was amplified from *E. coli* O157:H7 EDL933 using the primers 184-lacZ-F and 184-lacZ-R. The amplified *lacZ* gene was ligated to pACYC184 and transformed into *E. coli* DH5α to obtain the recombinant p184-*lacZ* plasmid. The *Eco*RI site of *lacZ* in p184-*lacZ* was mutated using the primers lacZ-EcoRI-Mu-F and lacZ-EcoRI-Mu-R. *lacZ* was sequenced using primers 184-CHK-F and 184-CHK-R. The 600-bp region upstream of *ehxC* in *E. coli* O157:H7 EDL933 was amplified using primers Gb1-PehxC-F and Gb1-PehxC-R (P_ehxC_). The 5' UTR of *ehxB* and its early coding region were amplified from 200 bp upstream the gene to the first 30 bp of the coding region using primers Gb2-UTRehxB-F and Gb2-UTRehxB-R (UTR_ehxB_). The P_ehxC_ and UTR_ehxB_ were inserted into p184-*lacZ* to obtain plasmid p184-P_ehxC_-UTR_ehxB_::*lacZ*. For complementation of *ehxB*, the gene and its UTR were amplified using primers Gb2-UTRehxB-F and Gb2-ehxB-R; P_ehxC_ with the obtained fragment was inserted into pACYC184, carrying restriction sites *EcoR*I and *Sca*I, to obtain plasmid pEHXB. All plasmids mentioned above were constructed using ClonExpress II One Step Cloning Kit (Vazyme, Nanjing, China; #C112) according to the manufacturer’s recommendations.

UTR_ehxA_ (amplified by UTRehxA-F and UTRehxA-R) and UTR_hlyE_ (amplified by UTRhlyE-F and UTRhlyE-R) were used to replace UTR_ehxB_ of p184-P_ehxC_-UTR_ehxB_::*lacZ* at *Spe*I and *Xho*I restriction sites to construct p184-P_ehxC_-UTR_ehxA_::*lacZ* and p184-P_ehxC_-UTR_hlyE_::*lacZ* respectively. Plasmids p184-P_ehxC_-UTR_ehxB_::*lacZ*, p184-P_ehxC_-UTR_hlyE_::*lacZ*, and p184-P_ehxC_-UTR_ehxA_::*lacZ* were used for the β-galactosidase assay. Site-directed mutagenesis was conducted at Genewiz Co., Ltd. (Suzhou, China). The GGA motifs in the DNA sequences of UTR_ehxB_ and UTR_hlyE_ harboring potential CsrA binding were mutated to TTC. P_ehxC_ with each mutated UTR was inserted into p184-*lacZ* carrying *EcoR*I and *Nco*I restriction sites to obtain the various mutant plasmids using the ClonExpress II One-Step Cloning Kit (Vazyme; #C112).

### CsrA binding sites prediction and *in silico* analysis

The genes containing multiple CsrA binding sites were predicted using the sequence-based algorithm reported by Kulkarni et al. [43]. Two hundred twenty-five potential targets were obtained from the EHEC genome, including *ehxB*. We also analyzed genes containing a potential single CsrA binding site. The potential CsrA target sequences were predicted to form secondary structures using the tools available on the mfold and CentroidFold web servers [44,45].

### Hemolytic activity assay

Hemolytic activity was detected using a washed blood agar plate (EHX plate) containing 3% washed sheep erythrocytes (Bersee, Beijing, China; #RCB001), 0.5 g/L sodium chloride, 10 mM CaCl_2_, 5 g/L yeast extract, and 10 g/L tryptone. Bacterial cells were cultured in LB medium overnight, collected by centrifugation, washed once with a fresh LB medium, and resuspended in the same medium to obtain optical density at 600 nm (OD_600_) = 0.8. Two microliters of the bacterial suspension were spotted on the EHX blood agar plate. The size of the hemolytic zone produced by each strain was recorded after incubation at 37°C for 24 h.

### Secreted protein preparation and EhxA detection

EHEC and its derived mutants were cultured overnight, collected, and washed twice. The strain was inoculated in a fresh LB at 1:100 dilution for further growth at 37°C When the OD_600_ of the sample reached 0.4, isopropyl β-d-1-thiogalactopyranoside (IPTG, 0.5 mM) was added while shaking at 250 rpm for 0.5 h at 37°C to induce the expression of _6His_EhxA. The sample was centrifuged at 10,000 ×*g* for 15 min, and the resulting supernatant was passed through a 0.22 μm filter, precipitated with 10% trichloroacetic acid (TCA) overnight and centrifuged at 20,000 ×*g* for 30 min. The precipitate was washed twice with cold acetone and dried in a fume hood. The pellet was diluted in 500 μL phosphate-buffered saline (PBS, 20 mM, pH 7.4) and stored at −20°C for further analysis.

For EhxA detection, western blotting was performed according to the procedures described by Wan et al. [46]. The membranes were probed with anti-His tag antibody (BBI, Shanghai, China; #D191001; 1:3000 dilution) overnight at 4°C and HRP-conjugated goat anti-mouse IgG (BBI; #D110087) as the secondary antibody (1:5000 dilution).

### β-Galactosidase assay

To examine the expression effects of *ehxB-lacZ* and *hlyE-lacZ* translational fusions, β-galactosidase assays were performed using *o*-nitrophenyl-*β*-D-galactopyranoside (ONPG) [47]. A single bacterial colony in 5 mL LB was cultured overnight with the corresponding antibiotics. The overnight culture was transferred to a fresh medium (1:100 dilution) and sub-cultured to the OD_600_ value that is optimal for investigation. The culture samples were prefilled with Z buffer and assayed using the method of Aviv and Gal-Mor [47].

### Expression and purification of the CsrA protein

CsrA expression was obtained as described by Sun et al. [48]. To construct the expression vector pET-*csrA*, *csrA* was amplified and cloned into pET28a carrying *Nco*I and *Xho*I sites to enable the production of CsrA containing a His-tag at its *N*-terminus (_6His_CsrA) (Supplementary material, Table S1). The plasmid was transformed into *E. coli* BL21 (DE3) (Tsingke, Beijing, China; #TSC-E01) to allow _6His_CsrA overexpression. The strain was cultured to OD_600_ = 0.5 at 37°C and 0.5 mM IPTG was then added while shaking at 250 rpm for 4 h at 37°C to induce _6His_CsrA expression. The same methods were applied to *E. coli* BL21 (DE3) cells harboring the pET28a vector as a control. The cells were collected by centrifugation at 5000 *g*, 4°C for 10 min, and frozen at −20°C until further use. CsrA purification was performed using the method described by Andrade et al. [29]. Protein concentration was determined using a Pierce™ BCA Protein Assay kit (Thermo Fisher Scientific, Waltham, MA, USA; # 23,227). The purified sample was stored at −20 °C.

### RNA electrophoretic mobility shift assay (RNA-EMSA)

RNA-EMSA was carried out using the method of Andrade et al. [29] with slight modifications. 5'-end-FAM-labeled RNA oligonucleotides encoding *ehxB* and *hlyE* leader sequences, which contain potential GGA motifs, were designed based on their RNA secondary structure prediction. All RNA oligonucleotides including positive control R9–43 and negative control *hns* were synthesized by Genewiz Co. Ltd. (Suzhou, China) to test for interactions with _6His_CsrA (Supplementary materials, Table S3) [34,49]. The binding assays for the gradient _6His_CsrA protein and 1 μM FAM-labeled RNA were performed according to Andrade et al. [29]. Signal bands were visualized using the Fusion FX fluorescence imaging instrument (VILBER, Paris, France).

Competition assays were used to calculate the apparent equilibrium binding constant (K_d_) of the CsrA-RNA complex. The concentration of RNA probes was 1 μM. The concentrations of CsrA were 0, 0.9, 1.8, 3.6, 7.2, 14.4, 28.8, 43.2, 57.6, and 72 nM. The CsrA-RNA binding curve was determined by CsrA concentration and shift band intensity in RNA-EMSA. K_d_ was evaluated using ImageJ software [50], following the manufacturer’s protocol.

### mRNA stability assay

The assay for mRNA stability was performed according to Andrade et al. [29] with slight modifications. For the detection of *ehxB*, EHEC wild-type and *ΔcsrA* mutant strains can be directly used in mRNA stability assays. For the detection of *hlyE*, both strains should be transformed into the pHLYE plasmid to increase the *hlyE* copy number for mRNA detection. Briefly, EHEC and its derived mutants were cultured overnight. The strain was inoculated in a fresh LB medium (1:100 dilution) for further growth at 37°C When OD_600_ reached 0.4, rifampicin (200 μg/mL) was added to inhibit transcription. Bacterial cultures were collected at 0, 2, 4, 6, 8, and 15 min after rifampicin treatment. The cells were harvested, and TRIzol (Invitrogen, Waltham, MA, USA; #15596026) was added for further treatment [29]. RNA extraction was performed using the RiboPure^TM^ Bacteria kit (Invitrogen; #AM1925), and RNA was quantified using Nanodrop One (Thermo Fisher Scientific; #ND-ONE-W) according to the manufacturer’s instructions. The purified RNA was used to obtain cDNA by reverse-transcription PCR (Takara Bio Inc., Kusatsu, Shiga, Japan; #RR014A). The amount of mRNA was detected using agarose gel electrophoresis [29]. To further detect the ratio of the transcript degradation in the wild type and *ΔcsrA* strains, quantitative real-time PCR (qPCR) was employed according to the method described by Wan et al. [46]. The reactions were performed using SYBR Premix Ex Taq II (Takara Bio Inc.; #RR820A) in the QuantStudio^TM^ 3 Real-Time PCR System (Applied Biosystems, Waltham, Ma, USA; #A28567).

### Statistical analysis

GraphPad Prism software (version 6.0; GraphPad Software, Inc., La Jolla, CA, USA) was used for statistical analysis. The results are expressed as the mean of the replicate measurements ± standard deviation (SD). A *P*-value of less than 0.05 indicated statistical significance in all tests performed.

## Results

### CsrA plays a central role in the repression of plasmid-coded hemolysis in EHEC

**Figure 1.**
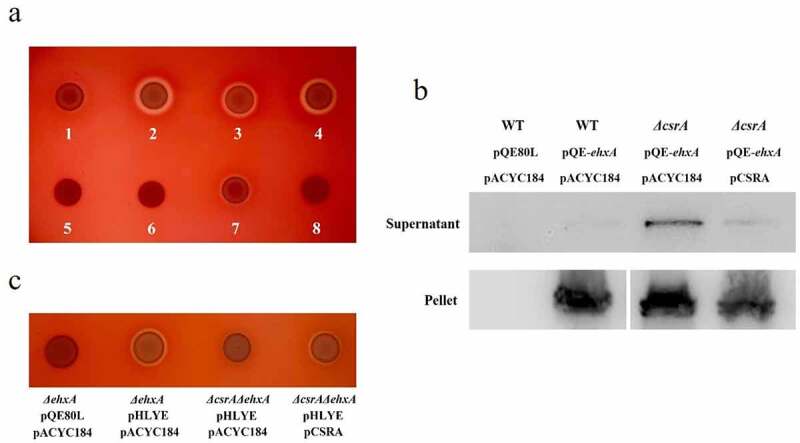
EHEC hemolysis regulation by CsrA.

To study the involvement of CsrA on the hemolytic activity of EHEC, a truncated *csrA* mutant with kanamycin resistance was constructed to reduce its RNA binding affinity and retain partial activity [42]. We first used a standard sheep blood agar plate to investigate the hemolytic activity of various EHEC strains (Supplementary material, Fig. S1). Similar to the previous reports [2], wild-type EHEC did not form an observable hemolytic circle in the standard blood agar plate. However, the *ΔcsrA* mutant strain showed a clear hemolytic circle, indicating that CsrA may downregulate hemolysis in EHEC (Supplementary material, Fig. S1a-S1d). We used an EHX blood agar plate to investigate CsrA-regulated hemolysis in EHEC. As evidenced in Figure 1(a), the wild-type EHEC formed a weak hemolytic circle on the EHX blood plate after 18 h incubation at 37°C. The *ΔcsrA* EHEC formed a large, clear hemolytic circle but it was reduced after complementation by a low-copy number plasmid with *csrA*, thus indicating that CsrA represses EHEC hemolysis. A similar, large hemolytic circle was formed by *ΔcsrA/ΔhlyE* while *ΔcsrA/ΔehxA* showed no evident hemolytic activity. This observation suggested that, in laboratory conditions, EHEC plasmid-borne hemolysin EhxA has a major hemolytic role, unlike HlyE. EhxB is a translocator of the EhxA secretion system [5]. The *ΔehxB* strain does not form a hemolytic circle by itself, but its hemolytic activity can be rescued by a plasmid copy of *ehxB* complementation, suggesting that like HlyB [4], EhxB is required for EhxA secretion and hemolytic activity in EHEC (Figure 1(a)).

a, Hemolytic effect of EhxA on EHX blood agar plate: 1, EHEC wild type; 2, *ΔcsrA*; 3, *ΔcsrA*/pCSRA; 4, *ΔcsrA*/*ΔhlyE*; 5, *ΔcsrA*/*ΔehxA*; 6, *ΔcsrA*/*ΔehxB*; 7, *ΔcsrA*/*ΔehxB*/pEHXB; 8, *ΔcsrA*/*ΔehxB*/pACYC184. b, EhxA secretion level of EHEC strains. Western-blotting analysis for the supernatant and pellet of EHEC culture. The target protein EhxA was added a His-tag at its N-terminus. The strains were transformed into pQE-*ehxA* and pCSRA. Empty plasmids pQE80L and pACYC184 were used as negative controls. c, Hemolytic effect of plasmid-copy HlyE on EHX blood agar plate.

Considering the high similarity of the intensively studied *E. coli* hemolysin cluster *hlyCABD* with *ehxCABD*, we evaluated the effect of CsrA on the hemolytic activity of uropathogenic *E. coli* (UPEC). We found that the hemolytic activity of UPEC *ΔcsrA* was similar to that of the wild-type, indicating that unlike that observed for *ehxCABD* from EHEC, CsrA had no obvious effect on *hlyCABD* regulation (Supplementary material, Fig. S1e and S1f). A similar phenotype of a classical repeats-in-toxin (RTX) leukotoxin (β-hemolysis, coded by *ltxCABD*), which is secreted by *Aggregatibacter actinomycetemcomitans*, a bacterial pathogen suspected to be involved in localized aggressive periodontitis and endocarditis, was also investigated [51] (Supplementary material, Fig. S1g and S1h). Our results indicated that CsrA might specifically regulate *ehxA*-derived hemolysis in EHEC.

### CsrA represses *ehxA*-derived hemolysis by interrupting EhxA secretion

EhxA is secreted by a classical T1SS in EHEC [5]. Hence, we hypothesized CsrA may be involved in the regulation of EhxA secretion. We constructed the plasmid pQE-*ehxA* to overexpress _6His_EhxA and detected _6His_EhxA secretion in the supernatant. The large amount of _6His_EhxA detected in the pellets of different bacterial strains indicated _6His_EhxA can be successfully overexpressed in EHEC (Figure 1(b)). However, the signal of the target protein was very weak in the EHEC wild-type supernatant, suggesting low _6His_EhxA secretion in the extracellular space (Figure 1(b)). The protein level of _6His_EhxA in the *ΔcsrA* strain supernatant was significantly higher than that in the wild-type and it was reduced by complementation of *csrA* in EHEC (Figure 1(b)). The significant amount of intracellular _6His_EhxA in the wild type and *ΔcsrA* strains further suggested CsrA represses _6His_EhxA secretion. Overall, these results demonstrate CsrA represses *ehxA*-derived hemolysis by interrupting EhxA secretion.

### Deletion of *csrA* impairs *hlyE*-derived hemolysis

EHEC *ΔcsrA/ΔehxA* showed no hemolytic activity on EHX blood agar plates, suggesting the plasmid pO157, which comprised the *ehxCABD* gene cluster, could play a major role in hemolysis (Figure 1(a)). To verify *hlyE* regulation by CsrA, we constructed a pHLYE plasmid containing the *hlyE* gene and its promoter to increase both transcript copy number and protein levels. A clear hemolytic zone was observed in *ΔehxA-*harboring pHLYE on EHX blood agar plates (Figure 1(c)). The hemolytic activity was significantly reduced in the *ΔehxA/ΔcsrA* strain, but it could be recovered by complementing the plasmid with *csrA* (Figure 1(c)). These results suggest CsrA positively regulates *hlyE*-derived hemolysis.

### *In silico* prediction of potential CsrA binding sites in hemolysin-coding genes

**Figure 2.**
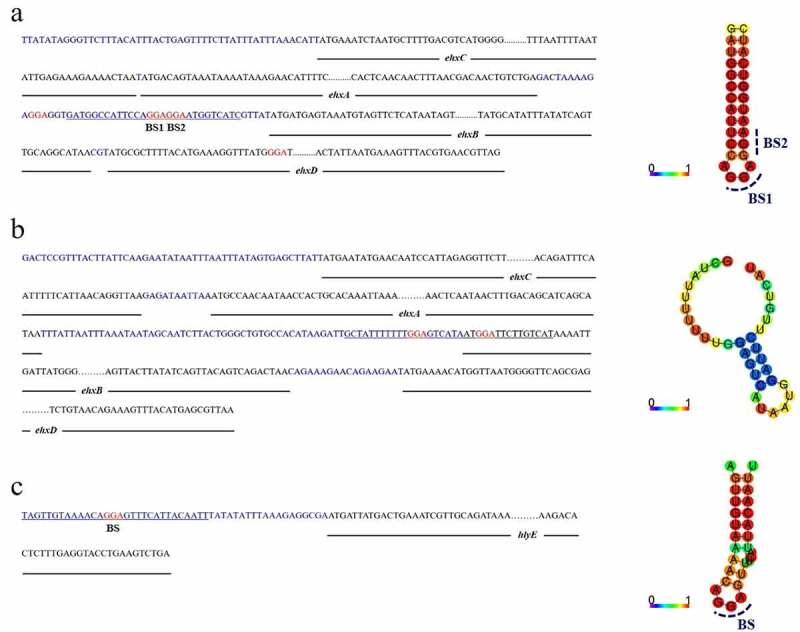
Overview of e*hxCABD* (EHEC), *hlyCABD* (UPEC), and *hlyE* (EHEC) operons.

CsrA normally binds to the 5' UTR or early coding region at sites containing a GGA motif that is often located in the single-stranded hairpin structure [30]. We found multiple GGA motifs in the 5' UTR of *ehxB* in the EHEC *ehxCABD* operon (Figure 2(a)). Binding site 1 (BS1) and binding site 2 (BS2) are located on the well-matched hairpin structure, which overlapped the *ehxB* Shine–Dalgarno (SD) sequence (Figure 2(a)). For the UPEC *hlyCABD* operon, two GGA motifs were found in the leader sequence of *hlyB*, but neither of them formed a well-matched stem-loop structure (Figure 2(b)), suggesting that this is not a potential CsrA-binding site. The transcription starting site of the *E. coli* general hemolysin coding gene *hlyE* has been described in a previous study [52]. We found only one GGA motif located on the loop of the hairpin structure at the 5' UTR of *hlyE* mRNA (Figure 2(c)), which is a potential CsrA-binding site and may be stabilized by CsrA (Figure 2(c)). Based on our sequence analysis and phenotype investigation, we inferred that CsrA might regulate both *ehxB* and *hlyE* in EHEC, with completely different implications in EHEC hemolysis.

The DNA sequences contain non-coding regions (blue), 30 bp of the 5’ and 3’ ends of gene coding regions (black), and GGA motifs (red). The secondary structure of underlined DNA sequences, predicted using CentroidFold, is displayed on the right side of each corresponding DNA sequence. a, *ehxCABD*; b, *hlyCABD*; c, *hlyE*.

### CsrA inhibits *ehxB* but activates *hlyE* expression *in* *vivo*

**Figure 3.**
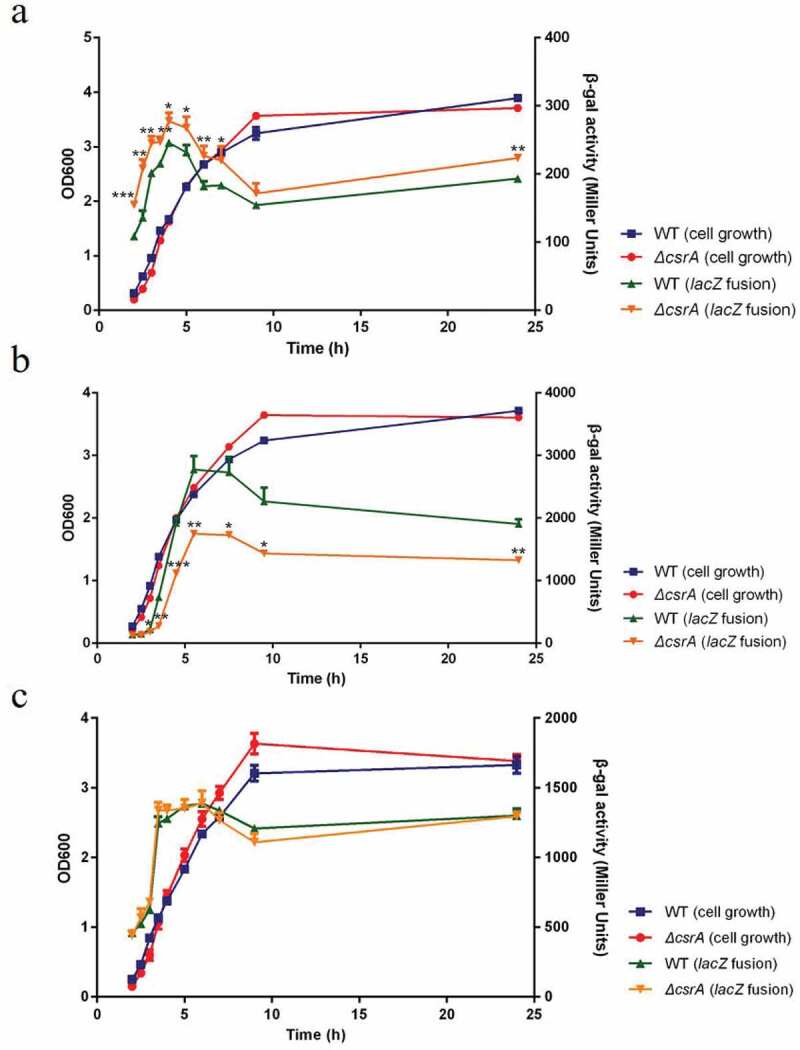
CsrA-Dependent regulation of *ehxB-lacZ*, *hlyE-lacZ*, and *ehxA-lacZ* translational fusion *in vivo*.

To determine the effects of CsrA on the expression of *ehxB* and *hlyE in vivo*, we used *ehxB-lacZ* and *hlyE-lacZ* translational fusions. We constructed low copy number plasmids that integrated P_ehxC_-UTR_ehxB_::*lacZ*, P_ehxC_-UTR_hlyE_::*lacZ*, and P_ehxC_-UTR_ehxA_::*lacZ*. Based on our prediction *in silico*, the *ehxA* gene should not be affected by CsrA and therefore P_ehxC_-UTR_ehxA_::*lacZ* was set as a negative control. The plasmids were electroporated into wild-type and *ΔcsrA* strains. The *ΔcsrA* strain grew slightly slower than the isogenic wild-type strain for 4 h but overtook it in the late exponential phase in LB medium (Figure 3). Expression of all fusion proteins was increased in the early exponential phase but decreased in the middle and late exponential phases (Figure 3). The *ehxB-lacZ* fusion expression in the *ΔcsrA* mutant showed a slightly higher activity compared to that in the wild-type strain throughout the exponential phase of the growth curve (Figure 3(a)). In this phase, the expression of *hlyE-lacZ* fusion was significantly higher in the wild-type than in the *ΔcsrA* strain (Figure 3(b)). No obvious difference in the expression of *ehxA-lacZ* fusion was found between the wild-type and *ΔcsrA* strains, indicating that CsrA does not directly affect *ehxA* expression (Figure 3(c)). These results demonstrate that CsrA represses *ehxB* expression but activates *hlyE* expression at the post-transcriptional level.

Bacterial β-galactosidase activity was detected at various time points throughout growth. Beta-galactosidase activity (green triangle: wild-type; orange triangle: *ΔcsrA*) and corresponding bacterial growth (blue square: wild-type; red circle: *ΔcsrA*) were determined for EHEC. a, *ehxB-lacZ* translational fusion; b, *hlyE-lacZ* translational fusion; c, *ehxA-lacZ* translational fusion. Each value represents the mean ± SD of three independent measurements. The significance of β-galactosidase activity in *ΔcsrA* compared with WT is also indicated: *p< 0.05, **p< 0.01, ***p< 0.001.

### CsrA directly interacts with the 5' UTR of *ehxB* and *hlyE*

**Figure 4.**
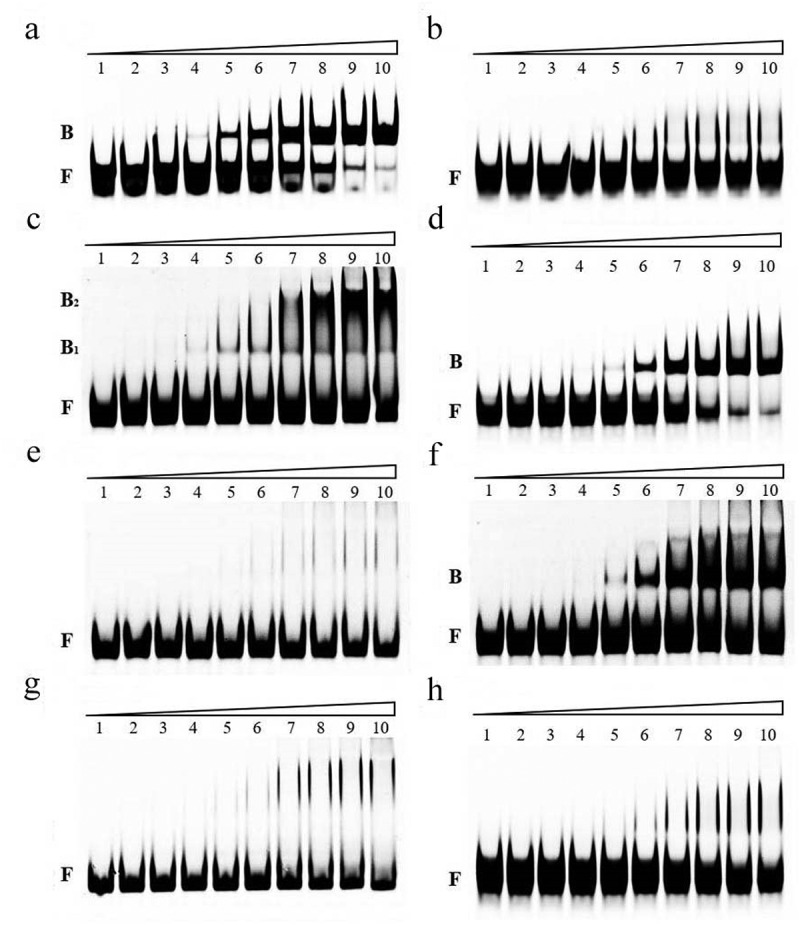
RNA mobility shift assays using various concentrations of purified _6his_CsrA on EHEC.

To investigate whether EHEC CsrA directly binds to the GGA motifs that were previously predicted in the leader sequence of *ehxB* and *hlyE* transcripts, RNA-EMSA was performed. R9–43 and the leader sequence of *hns* were used as positive and negative controls, respectively [34,49]. Our results demonstrate that EHEC _6His_CsrA promotes a band shift of the RNA probe R9–43, but not of the RNA probe *hns*, suggesting that the recombinant _6His_CsrA retains binding affinity (Figure 4(a,b)). The gel mobility shift analysis of the interaction between _6His_CsrA and FAM-labeled *ehxB* and *hlyE* leader probes was performed according to the binding reactions reported by Yakhnin et al. [53] The fraction of bound *ehxB* and *hlyE* increased proportionally to the concentration of _6His_CsrA (Figure 4(c,d)). The complex was observed at 3.6 nM _6His_CsrA for *ehxB* and 7.2 nM _6His_CsrA for *hlyE*, and most of the starting RNA was shifted at 72 nM _6His_CsrA. The apparent K_d_ values of *ehxB* and *hlyE* were 21.39 nM and 27.25 nM, respectively.

The concentration of CsrA in lanes 1 to 10 was 0, 0.9, 1.8, 3.6, 7.2, 14.4, 28.8, 43.2, 57.6, and 72 nM, respectively; the concentration of the FAM-labeled probe was 1 μM. a, interaction between _6His_CsrA and FAM-labeled positive control transcript R9-43; b, interaction between _6His_CsrA and FAM-labeled negative control transcript *hns*; c, interaction between _6His_CsrA and FAM-labeled transcript of *ehxB* (*ehxB*-Ori); d, interaction between _6His_CsrA and FAM-labeled transcript of *hlyE* (*hlyE*-Ori); e, interaction between _6His_CsrA and FAM-labeled transcript of *ehxB* with BS1 motif mutation (*ehxB*-MuBS1); f, interaction between _6His_CsrA and FAM-labeled transcript of *ehxB* with BS2 motif mutation (*ehxB*-MuBS2); g, interaction between _6His_CsrA and FAM-labeled transcript of *ehxB* with BS1 and BS2 motif double mutation (*ehxB*-MuBS1-2); h, interaction between _6His_CsrA and FAM-labeled transcript of *hlyE* with CsrA binding motif mutation (*hlyE*-Mu). B, bound RNA; F, free RNA.

### CsrA binding sites in the *ehxB* and *hlyE* transcripts

The RNA-EMSA results showed that CsrA has high affinity for *ehxB* and *hlyE* transcripts *in vitro* (Figure 4(c,d)). Mutation of the GGA motifs in potential binding sites to TTC was performed in accordance with our *in silico* predictions to investigate which site was crucial for CsrA interaction (Figure 2). Mutations in BS1 and BS2 (*ehxB*-MuBS1–2) abolished the binding affinity for *ehxB*. The binding activity of BS1 with a single mutation (*ehxB*-MuBS1) was similar to the activity of *ehxB*-MuBS1–2, but mutation of BS2 (*ehxB*-MuBS2) slightly reduced the activity compared to that of the wild-type probe (*ehxB*-Ori) (Figure 4(e,g)). These results demonstrate that the BS1 of *ehxB* transcript is essential for CsrA binding affinity. Only one GGA motif was located in the *hlyE* 5' UTR (Figure 2(c)). The *hlyE*-Ori probe exhibited a significant band shift (Figure 4(d)). However, no obvious band shift was observed for the CsrA and *hlyE*-Mu interaction, indicating that the motif located in the 5' UTR of *hlyE* was crucial for CsrA binding (Figure 4(h)).

**Figure 5.**
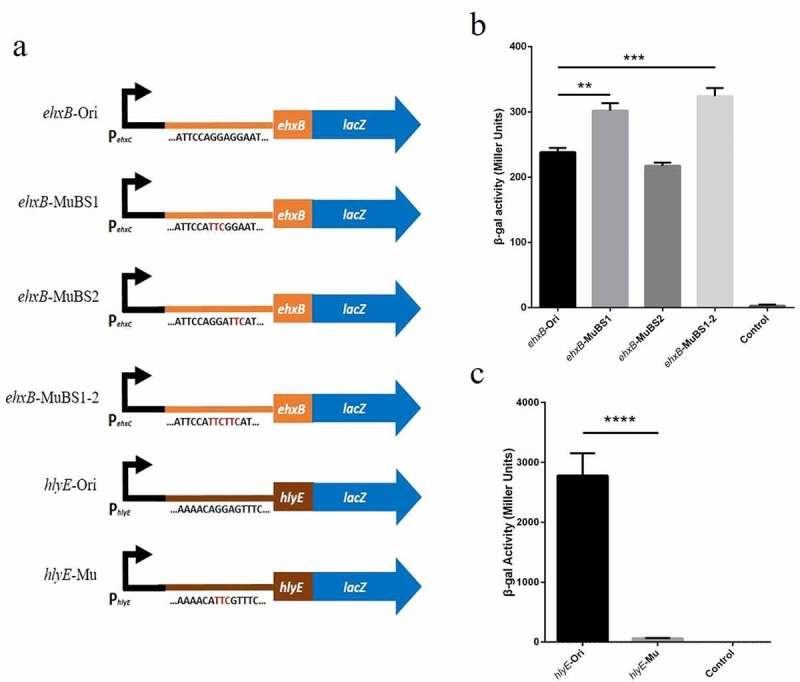
Effect of site-directed mutation in potential binding sites in the expression of *lacZ* translational fusion *in vivo*.

The same motif substitution (GGA to TTC) was used to construct the P_ehxC_-UTR_ehxB_::*lacZ* and P_ehxC_-UTR_hlyE_::*lacZ* translational fusions, and expression was detected using the ONPG method (Figure 5(a)). The *ehxB* expression of the double motif substitution strain was nearly 1.4-fold higher than that of the wild-type strain and that of the BS1 motif substitution strain was nearly 1.3-fold higher than that of the wild-type strain (Figure 5(b)). However, the expression of the wild-type leader sequence of *hlyE* was nearly 43-fold higher than that of the motif substitution strain (Figure 5(c)). These results are consistent with that of RNA-EMSA and confirm the presence of CsrA binding sites in *ehxB* and *hlyE* transcripts.

a, Schematic diagram of translational fusion constructs for β-galactosidase assays; b, *ehxB-lacZ* translational fusion detected by the ONPG method. Strains were cultured in LB medium for 5 h at 37 ℃ under constant shaking at 250 rpm; c, *hlyE-lacZ* translational fusion detected by the ONPG method. EHEC was cultured in LB medium for 5.5 h at 37 ℃ under constant shaking at 250 rpm. Significantly different β-galactosidase activity is indicated: **p< 0.01, ***p< 0.001, ****p< 0.0001.

### Effects of CsrA on *ehxB* and *hlyE* stability

**Figure 6.**
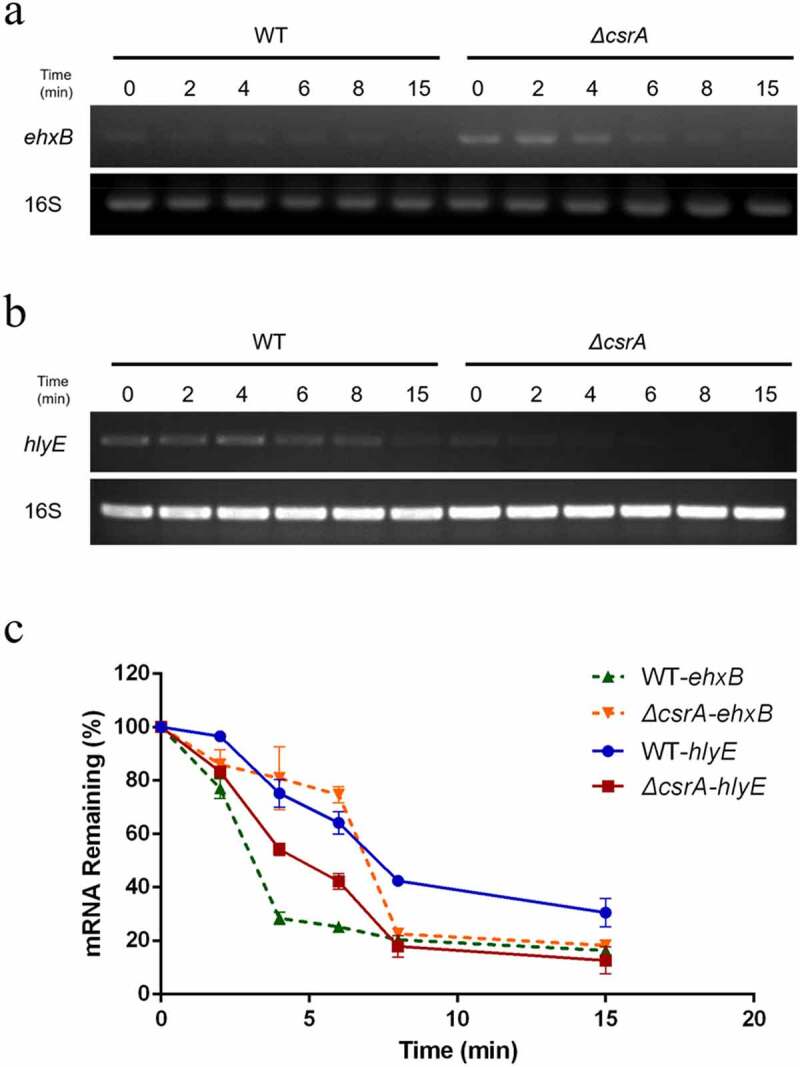
Stability of *ehxB* and *hlyE* mRNA as affected by CsrA in EHEC.

As demonstrated above, CsrA repressed *ehxA*-derived hemolysis and promotes *hlyE*-derived hemolysis on EHX blood agar plates (Figure 1(a,c)). The translational fusion and RNA-EMSA results demonstrated that CsrA directly binds to the 5' UTR of *ehxB* and leader region of the *hlyE* transcripts to affect their expression (Figure 4 and Figure 5). As translation efficiency can be influenced by mRNA stability [29], we examined the relative abundance of *ehxB* and *hlyE* transcripts using qPCR. Significantly more *ehxB* transcripts were found in *ΔcsrA* than in the wild-type, and their degradation rate in *ΔcsrA* was lower than that in the wild-type (Figure 6(a)). Conversely, significantly less *hlyE* transcripts were found in *ΔcsrA* than in the wild-type, and their degradation rate in *ΔcsrA* was higher than that in the wild-type (Figure 6(b)). We employed qPCR to analyze *hlyE* mRNA levels in the wild type and *ΔcsrA* strains. The *ehxB* transcript half-life in the wild-type was approximately 3.1 min, but in the *ΔcsrA* strain, it was approximately 7 min (Figure 6(c)). However, the *hlyE* transcript half-life in the wild-type was approximately 7.5 min, but in the *ΔcsrA* strain, it was approximately 4.7 min (Figure 6(c)). These results suggest that CsrA regulates *ehxB* and *hlyE* expression by affecting their mRNA stability.

EHEC wild-type and *ΔcsrA* cells were cultured in LB medium to OD_600_ = 0.4, treated with 200 μg/mL rifampicin, and collected at the indicated time points after treatment. Total RNA was extracted using the RiboPure^TM^ Bacteria kit, and the purified RNA was used for cDNA synthesis by reverse-transcription PCR. The cDNA was then used as template for further amplification. a, *ehxB* mRNA detection; b, *hlyE* mRNA detection. Amplifications were carried out for 29 cycles (*ehxB*) or 30 cycles (*hlyE*), and each product was analyzed by agarose gel electrophoresis. c, mRNA degradation ratio detection by qPCR. Dashed line: *ehxB*; green triangle: *ehxB* in wild-type; orange triangle: *ehxB* in *ΔcsrA*; solid line: *hlyE*; blue circle: *hlyE* in wild-type; red square: *hlyE* in *ΔcsrA*. 16S rRNA was used as the reference for normalizing the target products. Each value represents the mean ± SD of three independent measurements.

## Discussion

EhxA and HlyE are two hemolysins identified in EHEC [2,7]. According to our data, EhxA is the major contributor of EHEC hemolysis *in vitro*. Mutation of *csrA* led to the increased hemolytic activity of *ΔcsrA* strain on EHX blood agar plates compared to that of the wild type (Figure 1(a)). Other similar classical RTX toxins from bacterial pathogens, such as α-hemolysin HlyA from UPEC and leukotoxin LtxA (β-hemolysis) from *A. actinomycetemcomitans*, do not show different hemolytic activity between their wild type and *ΔcsrA* strains [51,54]. This indicates that CsrA may regulate *ehxA*-derived hemolysis specifically in EHEC.

To clarify how CsrA regulates *ehxA*-derived hemolysis, we overexpressed EhxA in various EHEC strains and examined its secretion. We observed that the level of secreted EhxA in the *ΔcsrA* strain was significantly increased compared to that in the wild-type but it was reduced by complementation with *csrA*. However, there was no obvious change in the levels of intracellular EhxA (Figure 1(b)). These results suggest that CsrA represses EhxA secretion.

The gene cluster *ehxCABD* encodes a classic bacterial T1SS and shares high similarity with *hlyCABD* in UPEC [5,7]. Based on genome screening and RNA secondary structure prediction, a well-matched stem-loop structure with two potential CsrA binding motifs was observed on the 5′ UTR of *ehxB*, which encodes the required translocator for T1SS (Figure 2(a)). However, no potential CsrA binding sites were found in the EHEC 5′ UTR of *ehxA* and UPEC *hlyCABD* gene cluster (Figure 2(a,b)). This led us to hypothesize that CsrA specifically regulates *ehxB*, not *ehxA*. Further β-galactosidase assays for detecting the activity of *ehxB-lacZ* and *ehxA-lacZ* translation fusion confirmed our prediction (Figure 3(a,c)).

**Figure 7.**
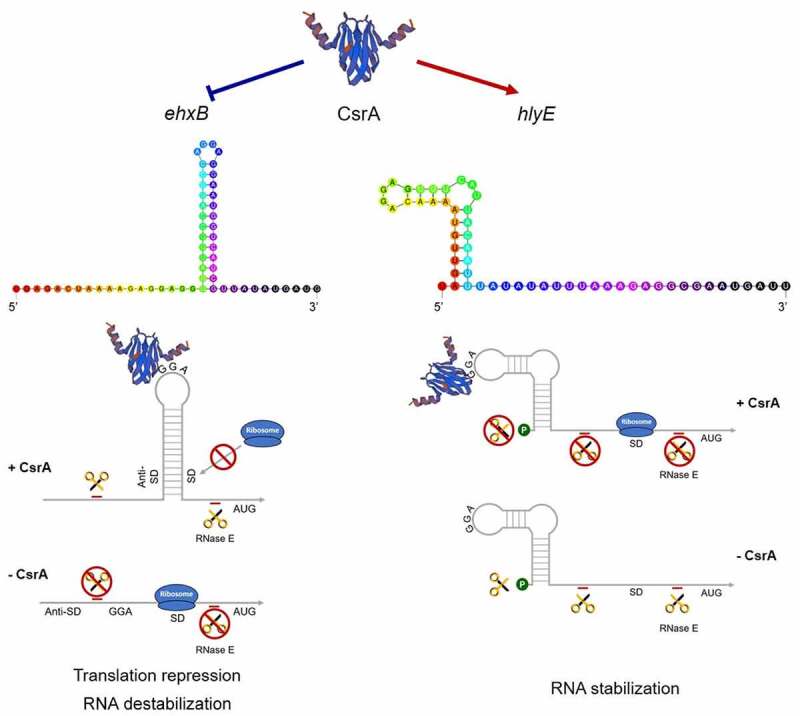
CsrA dual regulation of EHEC hemolysis.

RNA-EMSA revealed CsrA’s high affinity to the non-coding region of *ehxB* transcript. The BS1–2 double mutation, as well as the BS1 single mutation, completely abolished CsrA binding affinity, while the BS2 single mutation slightly reduced the binding activity (Figure 4(e-g)). These results indicate BS1 is crucial for the CsrA-*ehxB* interaction. Our hypothesis is further supported by the results of the β-galactosidase assay performed for detecting *ehxB-lacZ* translation fusion at the protein level, as CsrA specifically bound to the 5′ UTR of *ehxB* and repressed its translation (Figure 5(b)). The region containing the *ehxB* ribosome binding site may fold into a well-matched hairpin structure, and the CsrA binding site is located in the loop, blocking ribosome access and preventing translation (Figure 7). In addition, the RNA stability assay revealed CsrA promotes *ehxB* transcript degradation, indicating that CsrA may repress *ehxB* translation in two different manners. This proposed mechanism is similar to the regulation of *glgC* by CsrA [36].

Hemolysis regulation by CsrA was predicted in EHEC. The 5′ UTRs of both *ehxB* and *hlyE* can form well matched stem-loop structures with GGA motifs. CsrA directly binds to the RNA leader sequence of *ehxB* to repress its expression in two different ways: CsrA either binds to the SD sequence of *ehxB* to block ribosome access or to the *ehxB* transcript to promote its mRNA decay. It can also interrupt EhxA secretion and downregulate *ehxA*-derived hemolysis. Binding of CsrA to the *hlyE* transcript may stabilize its mRNA and promote *hlyE*-derived hemolysis.

HlyE is a pore-forming toxin unrelated to the RTX family. The gene is usually silent because it is strictly controlled by the global regulator H-NS at the transcriptional level [15]. We first demonstrated that CsrA directly regulates *hlyE* expression at the post-transcriptional level. The β-galactosidase assay for detecting *hlyE-lacZ* translational fusion confirmed the wild-type strain has significantly higher *hlyE*-derived hemolysis than the *ΔcsrA* strain (Figure 3(b)). There is only one GGA motif on the 5′ UTR of the *hlyE* transcript, which is located on the loop of the hairpin structure. Mutation of this motif led to significantly decreased *hlyE* expression (Figure 5(c)). Our results further showed that *hlyE* transcripts were less abundant in the *ΔcsrA* mutant than in the wild type and that *hlyE* transcript degradation was faster in the *ΔcsrA* mutant than that in the wild type (Figure 6). This indicates that CsrA upregulates *hlyE* expression by stabilizing *hlyE* mRNA. This is similar to the upregulation mechanism of the type III secretion system (T3SS) master regulator coding gene, *hrpG*, in *Xanthomonas citri*, and flagellum biosynthesis and chemotaxis master regulator coding gene, *flhDC*, in *E. coli* [29,38]. The latter mechanism is well-characterized in terms of *flhDC* regulation by CsrA in *E. coli*, with CsrA binding to the mRNA leader region and protecting it against 5′-end-dependent RNase E cleavage [38]. However, a recent report revealed that CsrA-upregulated *ymdA* expression not only stabilizes *ymdA* mRNA but also promotes 30S ribosome subunit binding [55]. In addition to RNA stabilization, CsrA could also bind to the leader region of *ymdA* mRNA, destabilizing the SD-blocking stem-loop structure and leading to the promotion of 30S ribosome subunit binding and translational activation [55]. In the present study, a single binding was found for the transcript of *hlyE*; it is located at the 5′-end of the leader sequence, far from the SD-sequence, and no other potential-binding motif was observed. Thus, *hlyE* regulation by CsrA seems to be mediated by RNA stabilization only (Figure 7). These results suggest a novel CsrA-mediated activation of *hlyE*-derived hemolysis in bacteria and elucidate the mechanism of *hlyE* regulation at the post-transcriptional level.

Our results also suggest that CsrA plays a dual role in the regulation of hemolytic activity. The global regulator CsrA normally acts as an organizer to switch metabolic pathways during the bacterial life cycle. Fine-tuning of CsrA activity allows bacterial pathogen to overcome many challenges in the host and promote pathogenicity. In *Pseudomonas aeruginosa*, the switch between acute and chronic infections is controlled by RsmA, a CsrA homolog [30]. It positively regulates acute virulence factors such as type IV pili and T3SS, and negatively regulates chronic virulence factors, such as biofilm formation and the type VI secretion system (T6SS) [30,56]. CsrA also regulates the switch from replicative to transmissive virulence phases of infection in *Legionella pneumophila* [30]. We hypothesize that CsrA regulates a switch between two hemolytic activities in EHEC. It negatively regulates *ehxA*-derived hemolysis and positively regulates *hlyE*-derived hemolysis. Results of the present study show that CsrA represses *ehxA*-derived hemolysis by interrupting its secretion but it activates *hlyE*-derived hemolysis by stabilizing its mRNA (Figure 1 and Figure 6). CsrA specifically binds to the *ehxB* 5′ UTR to repress EhxB expression and, in turn, blocks T1SS function. The suggested mechanism of *ehxB* repression is that CsrA prevents *ehxB* translation initiation by blocking ribosome access and promoting mRNA decay. The mechanism of *hlyE* activation is that CsrA specifically binds to the 5′-end of the transcript and reduces mRNA degradation by protecting it from hydrolysis by RNase (Figure 7). The two-component system (TCS) BarA/UvrY is a primary regulator of the Csr regulatory system of *E. coli* and is activated by short-chain fatty acids (SCFAs) or Krebs cycle intermediates [30,57]. In the intestinal tract, abundant SCFAs may activate the TCS BarA/UvrY during EHEC colonization and infection [30]. The activated TCS positively regulates expression of sRNA CsrB/C, leading to a decrease of free-CsrA in bacteria [30]. Lacking of free-CsrA upregulates *ehxB* expression, and in turn promotes *ehxA*-derived hemolysis. Meanwhile, the *hlyE*-derived hemolysis is downregulated due to its mRNA destabilization. The high level of secreted EhxA may promote cell damage via pore formation and cause apoptosis via the capase-9-dependent pathway [5,10]. Conversely, if the sRNA CsrB/C expression is repressed or hydrolyzed by RNase, more CsrA will be released. This abundance of free-CsrA may inhibit *ehxA*-derived hemolysis by repressing *ehxB* expression. On the contrary, *hlyE*-derived hemolysis will be upregulated because CsrA prevents its mRNA decay. This effect may promote EHEC to invade epithelial cells [20]. The circuit may assist EHEC to regulate the switch of *ehxA-* and *hlyE*-derived hemolysis according to different signals in the host’s intestinal tract or environment and perform distinct functions. Many factors function through or have connection with the BarA/UvrY, indicating the regulation circuits are more complex [30].

In the present study, the regulatory mechanism of EHEC hemolysis at the post-transcriptional level is reported for the first time. According to our results, the global regulator CsrA represses *ehxA*-derived hemolysis and activates *hlyE*-derived hemolysis, therefore regulating the switch between these two hemolytic activities. This reverse regulation effect of CsrA suggests that EhxA and HlyE may have other functions in bacterial metabolism and pathogenicity. Therefore, future studies should focus on comprehensively understanding the functions of EHEC hemolysins.

## Supplementary Material

Supplemental Material

## Data Availability

The authors confirm that the data supporting the findings of this study are available within the article and its supplementary materials.
