# Supplementary material for: Dual role of CsrA in regulating the hemolytic activity of Escherichia coli O157:H7
Source: Virulence. 2022 May 24;13(1):859–74. doi: 10.1080/21505594.2022.2073023 (PMC9132389; doi:10.1080/21505594.2022.2073023)
Supplement: Supplemental Material [file KVIR_A_2073023_SM7184.zip › 4-Supl_Tables_20220422-R4.docx]

Supplementary Figure for

**Dual role of CsrA in regulating the hemolytic activity of *Escherichia coli* O157:H7**

Zhibin Sun^1,2^, Ning Zhou^1^, Wenting Zhang^1^, Yan Xu^2^, Yu-Feng Yao^1,3,4*^

1 Laboratory of Bacterial Pathogenesis, Department of Microbiology and Immunology, Institutes of Medical Sciences, Shanghai Jiao Tong University School of Medicine, Shanghai 200025, China.

2 Jiangsu Province Key Laboratory of Oral Diseases, Nanjing Medical University, Nanjing 210029, Jiangsu, China.

3 Department of Infectious Diseases, Shanghai Ruijin Hospital, Shanghai 200025, China.

4 Shanghai Key Laboratory of Emergency Prevention, Diagnosis and Treatment of Respiratory Infectious Diseases, Shanghai 200025, China.

^*^Address correspondence to: [yfyao@sjtu.edu.cn](mailto:yfyao@sjtu.edu.cn) (Yao YF).

Table S1 Bacterial strains and plasmids used in this study

| Strain or plasmid | Genotype or description | Source or reference |
| --- | --- | --- |
| Strains |  |  |
| *Escherichia coli* O157:H7 str. EDL933 | EHEC O157: H7 isolated in 1982 from polluted food, producing Stx | Lab stock |
| *E. coli* K12 str. MG1655 | F^-^ λ^-^ *rph-1* | Lab stock |
| *Escherichia coli* str. CFT073 | *E.coli* pyelonephritis isolate, P1 *pap*, P2 *pap*, UPEC | Lab stock |
| *Aggregatibacter actinomycetemcomitans* | ATCC 29523 | Lab stock |
| *E. coli* DH5α | *Δ(argF-lac)169*, *φ80dlacZ58(M15)*, *ΔphoA8*, *glnX44(AS)*, *λ-*, *deoR481*, *rfbC1*, *gyrA96(NalR)*, *recA1*, *endA1*, *thiE1*, *hsdR17* | Novagen |
| *E. coli* BL21 (λDE3) | *lon-11*, *Δ(ompT-nfrA)885*, *Δ(galM-ybhJ)884*, λDE3 [*lacI, lacUV5-T7 gene 1, ind1, sam7, nin5*], *Δ46*, [mal+]K-12(λS), *hsdS10* | Novagen |
| EHEC *ΔlacI-Z* | *lacI-Z* deletion mutant derived from EHEC strain EDL933 | This study |
| EHEC *ΔcsrA* | *csrA* truncation of EHEC strain EDL933, Kan^R^ | This study |
| EHEC *ΔcsrB* | *csrB* deletion mutant derived from EHEC strain EDL933, Cm^R^ | This study |
| EHEC *ΔlacI-ZΔcsrA* | *lacI-Z* and *csrA* double mutant derived from EHEC strain EDL933, Km^R^ | This study |
| EHEC *ΔehxA* | *ehxA* deletion mutant derived from EHEC strain EDL933, Cm^R^ | This study |
| EHEC *ΔehxB* | *ehxB* deletion mutant derived from EHEC strain EDL933, Cm^R^ | This study |
| EHEC *ΔhlyE* | *hlyE* deletion mutant derived from EHEC strain EDL933, Cm^R^ | This study |
| EHEC *ΔcsrAΔehxA* | *csrA* and *ehxA* double deletion mutant derived from EHEC strain EDL933, Km^R^/Cm^R^ | This study |
| EHEC *ΔcsrAΔehxB* | *csrA* and *ehxB* double deletion mutant derived from EHEC strain EDL933, Km^R^/Cm^R^ | This study |
| EHEC*ΔcsrAΔhlyE* | *csrA* and *hlyE* double deletion mutant derived from EHEC strain EDL933, Km^R^/Cm^R^ | This study |
|  |  |  |
| Plasmids |  |  |
| pET28a | Plasmid used for expressing the protein with T7 promoter, Km^R^ | Novagen |
| pQE80YX1 | Plasmid used for expressing the protein with T5 promoter, Ap^R^ | Lab stock |
| pQE80L | Negative control plasmid for using pQE80YX1 to express protein | Lab stock |
| pACYC184 | *P15A ori cat^R^/ tet^R^* | Lab stock |
| pUC19 | pUC vector for gene cloning | Lab stock |
| p184-*lacZ* | EHEC *lacZ* (*EcoR*I mutation) inserted in *EcoR*I and *Nco*I site; for gene regulation study, Tet^R^ | This study |
| pKD46 | *repA*101(ts) *bla* *araC P_araR_-Red* | Lab stock |
| pKD3 | Source of *cat* gene | Lab stock |
| pKD4 | Source of *kat* gene | Lab stock |
| pCP20 | Flp recombinase expression plasmid for select marker deletion, Amp^R^/Cm^R^ | Lab stock |
| pET-*csrA* | pET28a harboring *csrA* at *Nde*I and *Xho*I site, expressing N terminal His-Tag CsrA protein with IPTG | This study |
| pCSRA | pACYC184 harboring *csrA* and its upstream of 500bp at *Nco*I and *Sca*I sites, expressing the CsrA protein for complementation | This study |
| pEHXB | pACYC184 harboring *ehxB* and *ehxC* promoter, expressing the EhxB protein for complementation. | This study |
| pHLYE | pQE80YX1 harboring *hlyE* and its upstream of 500bp at *Afe*I and *Fse*I sites. | This study |
| pQE-*ehxA* | pQE80YX1 harboring *ehxA* at *Afe*I and *Fse*I sites. Plasmid used for overexpressing the EhxA with IPTG. | This study |
| p184-P_ehxC_-UTR_ehxA_::*lacZ* | pACYC184 harboring *ehxA*::*lacZ* transcriptional fusion with *ehxC* promoter and *ehxA* UTR (-200 to +30). | This study |
| p184-P_ehxC_-UTR_ehxB_::*lacZ* | pACYC184 harboring *ehxB*::*lacZ* transcriptional fusion with *ehxC* promoter and *ehxB* UTR (-200 to +30). | This study |
| p184-P_ehxC_-UTR_hlyE_::*lacZ* | pACYC184 harboring *hlyE*::*lacZ* transcriptional fusion with *ehxC* promoter and *hlyE* UTR (-200 to +30). | This study |
| p184-P_ehxC_-UTR_ehxB-MUBS1_::*lacZ* | Predicted CsrA binding site BS1 mutation of p184-P_ehxC_-UTR_ehxB_::*lacZ* | This study |
| p184-P_ehxC_-UTR_ehxB-MUBS2_::*lacZ* | Predicted CsrA binding site BS2 mutation of p184-P_ehxC_-UTR_ehxB_::*lacZ* | This study |
| p184-P_ehxC_-UTR_ehxB-MUBS1-2_::*lacZ* | Predicted CsrA binding sites BS1 and BS2 mutation of p184-P_ehxC_-UTR_ehxB_::*lacZ* | This study |

Table S2 Oligonucleotide primers used in this study

| Primer name | Sequence (5’ to 3’) | Description |
| --- | --- | --- |
| csrA-KO-F | TAAATGCCCCGAAGGAAGTTTCTGTTCACCGTGAAGAGATCTACCAGCGTATGATTGAACAAGATGGATT | For *csrA* mutant construction |
| csrA-KO-R | GAGACGCGGAAAGATTAGTAACTGGACTGCTGGGATTTTTCAGCCTGGATTCAGAAGAACTCGTCAAGAA | For *csrA* mutant construction |
| csrA-CHK-F | TATCTACAGAAAAACGGAGGT | PCR check for *csrA* truncation |
| csrA-CHK-R | TTGTGTATATCGGCTAAACTT | PCR check for *csrA* truncation |
| ehxA-KO-F | TTATGTCTGAAGTAAAAAACAGACAAGATTTTAATTTTAATATTGAGAAAGAAAACTAATGTGTAGGCTGGAGCTGCTTC | For *ehxA* mutant construction |
| ehxA-KO-R | TTACTCATCATATAACGATGACCATTCCTCCTGGAATGGCCATCACCTCCTCTTTTAGTCCATATGAATATCCTCCTTAG | For *ehxA* mutant construction |
| ehxA-CHK-F | GGATAGCTCCATTTGGACAC | PCR check for *ehxA* deletion |
| ehxA-CHK-R | ATTTCGCTGCCAGTAACCAT | PCR check for *ehxA* deletion |
| ehxB-KO-F | GCACAATATCATAATATAACTGTCAATGCTGAAACTATAAGGCATCAGTATAATACCCACGTGTAGGCTGGAGCTGCTTC | For *ehxB* mutant construction |
| ehxB-KO-R | TTTCCGACTCATAATCAAGAGCACTGGTTGCTTCATCAAAAATAAGAATTCGAGGATTTGCATATGAATATCCTCCTTAG | For *ehxB* mutant construction |
| ehxB-CHK-F | TTCTTCAGTTTCAGGGTTCG | PCR check for *ehxB* deletion |
| ehxB-CHK-R | ACGTTATGCCTGCAACTGAT | PCR check for *ehxB* deletion |
| hlyE-KO-F | TTTCATTACAATTTATATATTTAAAGAGGCGAATGATTATGACTGAAATCGTTGCAGATAGTGTAGGCTGGAGCTGCTTC | For *hlyE* mutant construction |
| hlyE-KO-R | TCAGACTTCAGGTACCTCAAAGAGTGTCTTCTTACCGTGTCTTTTCTGATACTCATTACACATATGAATATCCTCCTTAG | For *hlyE* mutant construction |
| hlyE-CHK-F | AATAGAAGCATTCGCCATAA | PCR check for *hlyE* deletion |
| hlyE-CHK-R | GCCATTTCGTAGCCCTGTTT | PCR check for *hlyE* deletion |
| lacIZ-KO-L-F | CCGGAATTCATGATGAACGCATAAACCTCCTG | The upstream of target box amplification for *lacI-Z* deletion |
| lacIZ-KO-L-R | CCCGAGCTCTGCGCGAGAAGATTGTGCACTGC | The upstream of target box amplification for *lacI-Z* deletion |
| lacIZ-KO-Cm-F | CCCGAGCTCGTGTAGGCTGGAGCTGCTTCG | Cat cassette of target box amplification for *lacI-Z* deletion |
| lacIZ-KO-Cm-R | CCCAAGCTTCATATGAATATCCTCCTTAGT | Cat cassette of target box amplification for *lacI-Z* deletion |
| lacIZ-KO-R-F | CCCAAGCTTTAATAACCGGGCAGGCCATGT | The downstream of target box amplification for *lacI-Z* deletion |
| lacIZ-KO-R-R | CCGCTCGAGCAGGAAACGCCAATAACATAC | The downstream of target box amplification for *lacI-Z* deletion |
| lacI-Z-CHK-F | ACGATGACGGCATTATTACCTC | PCR check for *lacI-Z* deletion |
| lacI-Z-CHK-R | CAAATACCCGCGTACCCTGTT | PCR check for *lacI-Z* deletion |
| lacZ-EcoRI-Mu-F | GCCCGTCAGTATCGGCGGAATTTCAGCTGAGCGCCGGTCGCTA | Sited directed mutation primer for lacZ *EcoR*I |
| lacZ-EcoRI-Mu-R | TAGCGACCGGCGCTCAGCTGAAATTCCGCCGATACTGACGGGC | Sited directed mutation primer for lacZ *EcoR*I |
| 184-lacZ-F | CCGGAATTCACTATGATTACAGATTCACTGGC | For p184-lacZ construction |
| 184-lacZ-R | CATGCCATGGTTATTTTTGACACCAGACCAA | For p184-lacZ construction |
| Gb1-PehxC-F | GCCCTGGGCCAACTTTTGGCGAAAATGAGACGTGAATTCGGTGGGTTGCCACCAAATGA | Fragment 1 for p184-PehxC-ehxB::lacZ construction by In-Fusion |
| Gb1-PehxC-R | TTAATATCATTGGCAACACTGGTGATATTAACCACTAGTAATGTTTAAATAAATAAGAA | Fragment 1 for p184-PehxC-ehxB::lacZ construction by In-Fusion |
| Gb2-UTRehxB-F | CATTTACTGAGTTTTCTTATTTATTTAAACATTACTAGTGGTTAATATCACCAGTGTTG | Fragment 2 for p184-PehxC-ehxB::lacZ construction by In-Fusion |
| Gb2-UTRehxB-R | TAAAACGACGGCCAGTGAATCTGTAATCATAGTCTCGAGACTATTATGAGAACTACATT | Fragment 2 for p184-PehxC-ehxB::lacZ construction by In-Fusion |
| UTRhlyE-F | GGACTAGTAATGAAAAATATCACCCGGC | The forward primer for *hlyE* 5’UTR with its early coding region amplification with *Spe*I and *Xho*I sites |
| UTRhlyE-R | CCGCTCGAGTTTATCTGCAACGATTTCAG | The forward primer for *hlyE* 5’UTR with its early coding region amplification with *Spe*I and *Xho*I sites |
| UTRehxA-F | GGACTAGTGAGGAAAAAGTATCCTTATG | The forward primer for *ehxA* 5’UTR with its early coding region amplification with *Spe*I and *Xho*I sites |
| UTRehxA-R | CCGCTCGAGTACTGTCATATTAGTTTTCT | The reverse primer for *ehxA* 5’UTR with its early coding region amplification with *Spe*I and *Xho*I sites |
| Gb2-ehxB-R | TCAGCACCTTGTCGCCTTGCGTATAATATTTGCCCATGGTTATGCCTGCAACTGATATA | Reverse primer for ehxB with its upstream of 200 bp amplification |
| 28a-csrA-F | GGAATTCCATATGCTGATTCTGACTCGTCG | For pET-*csrA* construction |
| 28a-csrA-R | CCGCTCGAGTTAGTAACTGGACTGCTGGGATT | For pET-*csrA* construction |
| 184-csrA-F | CAATATGGACAACTTCTTCGCCCCCGTTTTCACCATGGGGTTGACGATTTAAAAAATCA | For pCSRA construction |
| 184-csrA-R | AAAAAAATTACGCCCCGCCCTGCCACTCATCGCAGTACTTTAGTAACTGGACTGCTGGG | For pCSRA construction |
| 80-ehxA-F | AAAGAGGAGAAATTAAGCATGCACCACCACCACCACCACACAGTAAATAAAATAAAGAA | For pQE-*ehxA* construction |
| 80-ehxA-R | TGATGGTGATGCGATCCTCTTCAGACAGTTGTCGTTAAAG | For pQE-*ehxA* construction |
| 80-hlyE-F | TTTCACACAGAATTCATTAAAGAGGAGAAATTAAGCGCTCGCTGCATAGACTGCTGATT | For pHLYE construction |
| 80-hlyE-R | CTTAGTGATGGTGATGGTGATGCGATCCTCTGGCCGGCCTCAGACTTCAGGTACCTCAA | For pHLYE construction |
| 184-CHK-F | CCTGGTGTCCCTGTTGATAC | Check primer for pACYC184 |
| 184-CHK-R | TGCTTTCGAATTTCTGCCAT | Check primer for pACYC184 |
| 80-CHK-F | TGAGCGGATAACAATTTCAC | Check primer for pQE80YX1 |
| 80-CHK-R | TGAGCGGATAACAATTTCAC | Check primer for pQE80YX1 |
| T7-Pro | TAATACGACTCACTATAGGG | Check primer for pET28a |
| T7-Ter | TATGCTAGTTATTGCTCAG | Check primer for pET28a |
| M13-F | TTGTAAAACGACGGCCAGT | Check primer for pUC19/pMD18T |
| M13-R | GGAAACAGCTATGACCATG | Check primer for pUC19/pMD18T |
| qPCR-hlyE-F | CCGCAGATGGAGCATTGGAT | qPCR detection for *hlyE* |
| qPCR-hlyE-F | ACGCCCGCAGCAATAGAATA | qPCR detection for *hlyE* |
| qPCR-16S-F | CAGCCACACTGGAACTGAGA | qPCR detection 16S rRNA |
| qPCR-16S-R | GTGCTTCTTCTGCGGGTAAC | qPCR detection 16S rRNA |

Table S3 RNA probes used in this study

| Probe name | Sequence (FAM-5’ to 3’) |
| --- | --- |
| R9-43 | GGGAAUUCAACUCCAUCUAGGCACAAGGAUGUGCCAUAGUACUCAAGCU |
| *hns* | CCUCAACAAACCACCCCAAUAUAAGUUUGAGAUUACUACAAUGAGCGAAG |
| *ehxB*-Ori | GGUGAUGGCCAUUCCAGGAGGAAUGGUCAUCGUUAU |
| *ehxB*-MuBS1 | GGUGAUGGCCAUUCCAUUCGGAAUGGUCAUCGUUAU |
| *ehxB*-MuBS2 | GGUGAUGGCCAUUCCAGGAUUCAUGGUCAUCGUUAU |
| *ehxB*-MuBS1-2 | GGUGAUGGCCAUUCCAUUCUUCAUGGUCAUCGUUAU |
| *hlyE*-Ori | UUCAUUAAUAGUUGUAAAACAGGAGUUUCAUUACAAUUUAUAUAU |
| *hlyE*-Mu | UUCAUUAAUAGUUGUAAAACAUUCGUUUCAUUACAAUUUAUAUAU |
