# Supplementary material for: Dual role of CsrA in regulating the hemolytic activity of Escherichia coli O157:H7
Source: Virulence. 2022 May 24;13(1):859–74. doi: 10.1080/21505594.2022.2073023 (PMC9132389; doi:10.1080/21505594.2022.2073023)
Supplement: Supplemental Material [file KVIR_A_2073023_SM7184.zip › 5-Supl_Figures-20220422-R4.docx]

Supplementary Figure for

**Dual role of CsrA in regulating the hemolytic activity of *Escherichia coli* O157:H7**

Zhibin Sun^1,2^, Ning Zhou^1^, Wenting Zhang^1^, Yan Xu^2^, Yu-Feng Yao^1,3,4*^

1 Laboratory of Bacterial Pathogenesis, Department of Microbiology and Immunology, Institutes of Medical Sciences, Shanghai Jiao Tong University School of Medicine, Shanghai 200025, China.

2 Jiangsu Province Key Laboratory of Oral Diseases, Nanjing Medical University, Nanjing 210029, Jiangsu, China.

3 Department of Infectious Diseases, Shanghai Ruijin Hospital, Shanghai 200025, China.

4 Shanghai Key Laboratory of Emergency Prevention, Diagnosis and Treatment of Respiratory Infectious Diseases, Shanghai 200025, China.

^*^Address correspondence to: [yfyao@sjtu.edu.cn](mailto:yfyao@sjtu.edu.cn) (Yao YF).


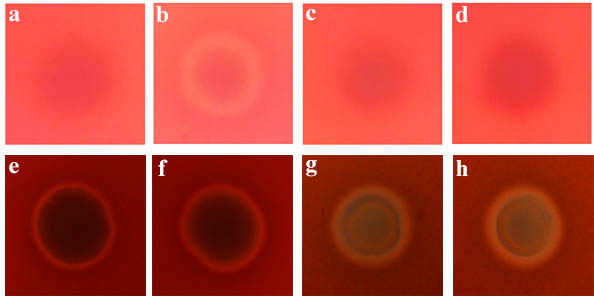


Fig. S1 Hemolytic activity of various bacterial strains on standard blood agar plate

The strains were inoculated to 5 mL LB (EHEC, UPEC and *E. coli* K12 MG1655) and BHI liquid media (*A. actinomycetemcomitans*) respectively and cultured overnight. Two-microliter of the bacterial suspension was spotted on the standard blood agar plate and cultured at 37℃ for EHEC and *E. coli* K12 MG1655 (48h), UPEC (24h) and *A. actinomycetemcomitans* (48h with 5% CO_2_). a-d, EHEC hemolysis analysis on standard sheep blood agar plate (photographed from plate back side); a, EHEC wild type strain; b, EHEC *ΔcsrA* strain; c, EHEC *ΔcsrB* strain; d, *E. coli* K12 MG1655. Compared EHEC wild type, EHEC *ΔcsrA* performed clear hemolytic zone. CsrB is a sRNA antagonist of CsrA. Mutation of CsrB showed no obvious change with wild type. *E. coli* K12 MG1655 is a negative control, and do not show hemolytic effect on standard blood agar plate. e-h, UPEC and *A. actinomycetemcomitans* hemolysis analysis on standard sheep blood agar plate (photographed from plate front side); e, UPEC wild type strain; f, UPEC *ΔcsrA* strain; g, *A. actinomycetemcomitans* wild type strain; h, *A. actinomycetemcomitans* *ΔcsrA* strain. Mutation of *csrA* showed no obvious change compared with their wild type strains in UPEC and *A. actinomycetemcomitans* respectively.
